# Transmyocardial laser revascularization versus medical therapy for refractory angina

**DOI:** 10.1590/S1516-31802011000300013

**Published:** 2011-05-05

## Abstract

**BACKGROUND:**

Chronic angina and advanced forms of coronary disease are increasingly more frequent. Although the improved efficacy of available revascularization treatments, a subgroup of patients present with refractory angina. Transmyocardial laser revascularization (TMLR) has been proposed to improve the clinical situation of these patients.

**OBJECTIVE:**

To assess the efficacy and safety of TMLR versus optimal medical treatment in patients with refractory angina in alleviating the severity of angina and improving survivorship and heart function.

**CRITERIA FOR CONSIDERING STUDIES FOR THIS REVIEW:**

We searched the Cochrane Central Register of Controlled Trials on The Cochrane Library (Issue 2 2007), MEDLINE (January 2006 to June 2007), EMBASE (2004 to June 2007) and ongoing studies were sought using the metaRegister of Controlled Trials database (mRCT) and ClinicalTrials.gov databases. No languages restrictions were applied. Reference lists of relevant papers were also checked.

**SELECTION CRITERIA:**

Studies were selected if they fulfilled the following criteria: randomized controlled trials of TMLR, by thoracotomy, in patients with angina grade III-IV who were excluded from other revascularization procedures. From a total of 181 references, 20 papers were selected, reporting data from seven studies.

**DATA COLLECTION AND ANALYSIS:**

Two reviewers abstracted data from selected papers. The reviewers performed independently both quality assessment and data extraction. Selected studies present methodological weaknesses. None of them fulfilled all the quality criteria.

**MAIN RESULTS:**

Seven studies (1137 participants of which 559 randomized to TMLR) were included. Overall, 43.8 % of patients in the treatment group decreased two angina classes as compared with 14.8 % in the control group, odds ratio (OR) of 4.63 (95% confidence interval (CI) 3.43 to 6.25), and heterogeneity was statistically significant. Mortality by intention-to-treat analysis at both 30 days (4.0 % in the TMLR group and 3.5 % in the control group) and 1 year (12.2 % in the TMLR group and 11.9 % in the control group) was similar in both groups. The 30-days mortality as treated was 6.8% in TMLR group and 0.8% in the control group, showing a statistically significant difference. The pooled OR was 3.76 (95% CI 1.63 to 8.66), because of the higher mortality in patients crossing from standard treatment to TMLR.

**AUTHORS’ CONCLUSIONS:**

There is insufficient evidence to conclude that the clinical benefits of TMLR outweigh the potential risks. The procedure is associated with a significant early mortality.

**José Henrique Andrade Vila, MD. Clinical Head of Heart Transplantation and the Intensive Care Unit of Professor Dr. José Pedro da Silva's team at Hospital Beneficência Portuguesa, São Paulo, Brazil.**

**José Pedro da Silva, MD. Cardiovascular Surgeon and Head of Team at Hospital Beneficência Portuguesa, São Paulo, Brazil.**

This is the abstract of a Cochrane Review published in the Cochrane Database of Systematic Reviews (CDSR) 2010, Issue 12, DOI: 10.1002/14651858.CD003712.pub1. (www.thecochranelibrary.com). For full citation and authors details see reference 1.

**For Latin America and the Caribbean, the full text is freely available from:** http://cochrane.bvsalud.org/cochrane/show.php?db=reviews&mfn=2037&id=CD003712&lang=pt&dblang=&lib=COC&print=yes

**For other regions, the abstract is available from:** http://onlinelibrary.wiley.com/o/cochrane/clsysrev/articles/CD003712/frame.html

**This section was edited under the responsibility of the Brazilian Cochrane Center**

## COMMENTS

We fully agree with the review authors’ conclusions^1^ that there is no proof in the literature that TMLR has clinical benefits that outweigh the potential risks. Like in the Vineberg procedure in the past, the distal perfusion through the microcirculation can be quite variable from patient to patient and intramyocardial hematomas may result, with serious consequences.

The more sophisticated techniques of epicardial myocardial revascularization, using internal thoracic arteries in sequential anastomosis to two or even three coronary arteries, or using saphenous veins in bypasses to reach truly distal portions of diseased arteries, and the use of other arterial conduits, usually eliminates angina in patients with good enough myocardial function. For patients with extremely severe and diffuse coronary artery disease or bad ventricular function, heart transplantation should be considered.
